# A comprehensive approach, based on the use of *Caenorhabditis elegans*, mouse, and human models, elucidates the impact of *Lactiplantibacillus plantarum* TWK10 on exercise performance and longevity

**DOI:** 10.1016/j.crfs.2025.101015

**Published:** 2025-03-06

**Authors:** Jian-Fu Liao, Chia-Chia Lee, Mon-Chien Lee, Han-Yin Hsu, Ming-Fu Wang, Chi-Chang Huang, San-Land Young, Koichi Watanabe, Jin-Seng Lin

**Affiliations:** a Culture Collection and Research Institute, SYNBIO TECH INC., No. 66, Beiling 6th Road., Luzhu District, Kaohsiung City, 821, Taiwan; b Graduate Institute of Sports Science, National Taiwan Sport University, No. 250, Wenhua 1st Road., Guishan District, Taoyuan City, 333325, Taiwan; c Center for General Education, Taipei Medical University, No. 250, Wuxing Street, Xinyi District, Taipei City, 110, Taiwan; d Department of Food and Nutrition, Providence University, No. 200, Section 7, Taiwan Boulevard, Shalu District, Taichung City, 43301, Taiwan; e Department of Animal Science and Technology, National Taiwan University, No. 50, Lane 155, Section 3, Keelung Road, Taipei City, 10672, Taiwan

**Keywords:** *Lactiplantibacillus plantarum* TWK10, *Caenorhabditis elegans*, Mice, Humans, Exercise performance, Longevity, Cross-species validation

## Abstract

The functionality of probiotics is highly influenced by culture and processing conditions, making batch stability validation through human or mouse trials impractical. Here, we employed a comprehensive approach using *Caenorhabditis elegans*, mouse and human models to elucidate the beneficial effects of *Lactiplantibacillus plantarum* TWK10 (TWK10). In *C. elegans*, TWK10 administration significantly prolonged lifespan by 26.1 ± 11.9 % (*p* < 0.05), enhanced locomotion (*p* < 0.01) and muscle mass (*p* < 0.001), elevated glycogen storage (*p* < 0.05), and reduced lipid accumulation (*p* < 0.001), outperforming *Lacticaseibacillus rhamnosus* GG and *L. plantarum* type strain ATCC 14917^T^. We also confirmed the equivalence of laboratory-prepared and mass-produced TWK10 in ergogenic efficacy using *C. elegans* assay. In mice, oral administration of mass-produced TWK10 significantly enhanced exercise performance and glycogen storage in muscle and liver in a dose-dependent manner. In a clinical study involving healthy male adults, significant improvements in grip strength (1.1-fold, *p* < 0.01) and exhaustion time (1.27-fold, *p* < 0.01), and significant reductions in circulating lactate and ammonia levels were observed in the TWK10 group (1 × 10^10^ colony-forming unit/day) compared to the control group. Both humans and mice receiving mass-produced TWK10 showed improved body composition with increased muscle mass and reduced fat mass. In conclusion, TWK10 demonstrates superior longevous and ergogenic effects in *C. elegans* compared to reference strains. The consistent ergogenic efficacy of mass-produced TWK10 across *C. elegans*, mice, and humans, highlights the utility of *C. elegans* as a reliable model for probiotic research and industrial application.

## Highlights

• Beneficial effects of TWK10 were verified in *C. elegans*, mice, and humans.
• Ergogenic effects of lab- and factory-produced TWK10 are equivalent.
• Cross-species analysis fully validated ergogenic effects of mass-produced TWK10.
• The 10 billion CFU/day dose of TWK10 provided sufficient ergogenic benefits.
• *C. elegans* is suitable for advanced probiotic research and industrial applications.

## Graphical abstract

**Figure:**
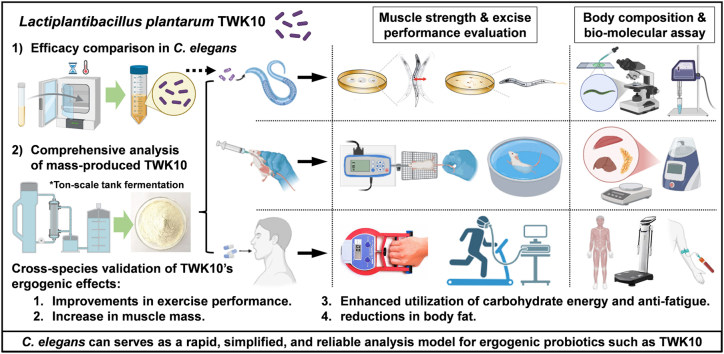

## 1 Introduction

Probiotics, with the potential to promote lifelong health and well-being, have evolved from fermented foods to essential health supplements supported by a robust and stable industry (Hill et al., 2014). Unlike chemical drugs with known molecular mechanisms, the beneficial mechanisms of probiotics often involve multiple pathways of the host including intestinal flora; nutritional metabolism; and the immune, endocrine, and nervous systems (Ansari et al., 2023). The numerous pathways through which probiotics influence host health allow for profound and systemic enhancement, including modulating neurological and mental health of host through brain-gut axis (Liu et al., 2016; Liao et al., 2019), alleviating inflammatory and degenerative disorders through strengthening the intestinal barrier (Napoles-Medina et al., 2023; Zheng et al., 2024), and influencing host development through immune pathways (Park et al., 2020; Lee et al., 2023). Since experimental conditions are far removed from the complex conditions of a host, most cellular models are insufficient for detailing the effects and operating mechanisms of functional strains. Therefore, live animal experiments are irreplaceable during the entire stage of functionality development, and rigorous clinical trials are necessary to substantiate relevant health benefits of probiotic strains (Papadimitriou et al., 2015; Torres-Maravilla et al., 2022).

Over the past century, probiotics have transitioned from their origins in fermented foods to becoming a vital health supplement produced by a robust and stable industry. Despite the extensive scientific evidence supporting the benefits of probiotics, significant discrepancies have been reported in the practical application of probiotics across different population groups. Moreover, substantial gap remains in the external environment, pressure, and reaction time experienced by probiotic strains between industrial-grade manufacturing and laboratory cultivation, which might in turn affect their gene expression and synthetic capacities (Bianchi et al., 2020; Zanni et al., 2017; Fenster et al., 2019). In addition to ensuring survival and stability, efficacy verification is also crucial for live probiotic products, especially as many optimization and adjustment operations evolve with technology and product development (Ahire et al., 2023). Nevertheless, efficacy confirmation by animal and human trials is particularly expensive, time-consuming and difficult to implement for industrial purposes. Thus, there is an urgent need for a validation platform, particularly one based on model organisms with a gastrointestinal tract (Lee et al., 2023; Bianchi et al., 2020; Zanni et al., 2017), to drive both research and industrial innovation in an effective and operationally efficient manner.

*Caenorhabditis elegans* is a bacterivorous nematode with an intact microbiota-gut-brain axis that is functionally and morphologically similar to humans, making it suitable for mechanistic investigation of gut microbiota and their effects on metabolism, development, behavior, pathogenesis, and lifespan (Kumar et al., 2020; Ortiz de Ora and Bess, 2021). The ease of achieving a germ-free state makes *C. elegans* ideal for verifying the direct effect of probiotics and their relevant substances on the host (Poupet et al., 2020; Urrutia et al., 2020). Considering the improvement of research efficiency and feasibility, many studies in probiotic research have utilized *C. elegans* as an initial *in vivo* approach before animal experiments (Marsova et al., 2020; Watanabe et al., 2020; Guantario et al., 2018; Veisseire et al., 2020; Lee et al., 2011; Wang et al., 2011). As a model organism, *C. elegans* also exhibits advantages of high fertility rates, short life cycle and well-studied genomics that enable high-throughput manipulations (Anderson et al., 2018; Manohar et al., 2022; Kirchweger et al., 2023). Once the efficacy characterization of a particular strain has been established, the integration of omics platforms with *C. elegans* analysis could serve as a powerful evaluation tool for industrial purposes (Bianchi et al., 2020; Zanni et al., 2017).

Previous studies have shown that *Lactiplantibacillus plantarum* TWK10 (TWK10) enhances exercise performance and muscle mass, and alleviates age-related cognitive decline in both mice and humans. Additionally, it changes body composition towards a healthy configuration and improves anti-fatigue capabilities, highlighting its unique potential as an ergogenic aid (Chen et al., 2016; Huang et al., 2019; Lee et al., 2021a, 2021b). *C. elegans* possesses motoneurons that innervate body wall muscles to perform locomotion behavior (Gjorgjieva et al., 2014), and its metabolic and physiological processes are similar to those of higher organisms (Watts and Ristow, 2017; Goyache et al., 2024; Gieseler et al., 2017). Nevertheless, studies investigating the ergogenic effects of supplements using *C. elegans* models remain limited. In this study, we analyzed the impact of TWK10 on *C. elegans*, with a particular focus on the ergogenic effects. *Lacticaseibacillus rhamnosus* GG (LGG), a strain with long-standing history (Capurso, 2019), and *L. plantarum* type strain ATCC 14917^T^ were simultaneously tested for efficacy comparison. Furthermore, to confirm whether the *C. elegans* model serves as a rapid, simplified, and reliable alternative analysis model for those performed in mice and humans, we conducted a comprehensive analysis to determine whether the ergogenic effects of industrially mass-produced TWK10 are equivalent in *C. elegans*, mice, and humans.

## 2 Materials and methods

### 2.1 Biomaterials overview

#### 2.1.1 *C. elegans*, bacterial strains and culture methods

The wild-type *C. elegans* N2 strain and *Escherichia coli* OP50 (OP50) were obtained from the Caenorhabditis Genetics Center (Minneapolis, MN, USA). The nematodes were cultivated at 15–20 °C in petri dishes on nematode growth medium (NGM; Agar 17.5 g/L, NaCl 3.0 g/L, peptone 2.5 g/L, Cholesterol 5 mg/L). Activated OP50 bacterial cells were grown in Tryptone Soy Broth (TSB; HiMedia, Kennett Square, PA, USA) at 37 °C for 16–18 h, and then 100–120 μL OP50 was dispensed and spread onto the NGM plate. The seeded OP50 was allowed to grow overnight at room temperature to form a bacteria lawn that served as a food source for the nematodes (Brenner, 1974). *L*. *plantarum* TWK10 (STCC 1008) was obtained from the Culture Collection and Research Institute of SYNBIO TECH INC. (Kaohsiung, Taiwan). *L*. *rhamnosus* BCRC 16000 (ATCC 53103, LGG) and *L*. *plantarum* BCRC 10069^T^ (ATCC 14917^T^; type strain) were obtained from the Bioresource Collection and Research Center (BCRC, Hsinchu, Taiwan). Lactic acid bacteria (LAB) were grown in de Man, Rogosa, and Sharpe broth (MRS; Merck, Darmstadt, Germany) at 37 °C for 16–18 h.

#### 2.1.2 Mass-produced TWK10

The impact of mass-produced TWK10 was investigated using two forms of the same substance: factory-produced TWK10 bulk bacterial powder (for *C. elegans* and animal studies) and the same substance in capsule form (for clinical study). Thawed TWK10 was activated multiple times to produce a liter-scale bacterial broth, and then inoculated into ultra-high-temperature sterilized formulated substrate (allergen-free) for ton-scale tank fermentation. Bacterial powder was prepared by mixing freeze-dried bacterial cells with the appropriate amount of maltodextrin to a concentration of 1 × 10^10^ colony-forming unit (CFU)/g and stored at −20 °C until use. For clinical trials, each labeled TWK10 capsule contained 5 × 10^9^ CFU lyophilized bacterial powder and was standardized with the mixture of maltodextrin (99%) and microcrystalline cellulose (1%).

### 2.2 *C. elegans* study

#### 2.2.1 *C. elegans* synchronization and LAB feeding

Sufficient amounts of egg-bearing nematodes were collected and washed twice with sterile water. L0 eggs were extracted in sodium hypochlorite-potassium hydroxide solution and then incubated in M9 buffer (3 g/L KH_2_PO_4_, 6 g/L Na_2_HPO_4_, 5 g/L NaCl, 100 mM MgSO_4_) at room temperature overnight for synchronization. The administration of LAB to synchronized L1 stage larvae was performed following previously published methods with slight modifications (Zhang et al., 2022; Yun et al., 2021). Cryopreserved LAB were activated twice in MRS and activated OP50 bacterial cells were grown in TSB at 37 °C for 16–18 h, and then collected by centrifugation at 8000×*g* for 5 min. Bacterial pellets were washed twice with M9 buffer, and then adjusted to 1 × 10^10^ CFU/mL according to the optical density at 600 nm. For mass-produced TWK10 validation, four factory batches of bacterial powder (BP01 – 04), which were produced using identical manufacturing processes within 1 year, were randomly selected. Next, 1 g bacteria powder was dissolved in 1 mL M9 buffer (1 × 10^10^ CFU/mL). A 500 μL aliquot of each bacterial cell (5.0 × 10^9^ CFU total) was individually spread on OP50-seeded NGM plates. Synchronized L1 stage larvae were subsequently dropped on LAB-OP50-seeded NGM plates and cultured at 20 °C to obtain young adult (Day 1) and mature adult (Day 5) nematodes. 5-Fluro-2ʹ-deoxyuridine (FudR, 200 μM) in M9 buffer was added at L4 stage to prevent offspring interference.

#### 2.2.2 Lifespan analysis

The lifespans were determined using a liquid culture method with some modifications (Lionaki and Tavernarakis, 2013). Briefly, N2 wildtype nematodes of all experimental groups were individually transferred with a platinum wire to a 48-well plate (16–20 worms per well, 100–120 worms per group) containing S medium with OP50 (≒10^8^ CFU/mL) and FudR (200 μM). The 48-well plates were incubated at 25 °C, and the numbers of worms alive, dead or censored in each well were recorded every 2 days until the last nematode perished. The survival curves for each strain-fed groups were determined using the Kaplan–Meier method, and significant differences among group were analyzed using the log-rank (Mantel–Cox) test. The mean life span (MLS) of nematodes in each group was estimated as previously described (Wu et al., 2006; Komura et al., 2022).

#### 2.2.3 Locomotion analysis

The crawling (Park et al., 2008) and swimming (Wang et al., 2019) locomotion assays were performed following previously published methods with slight modifications. Briefly, the nematodes of each experimental group were individually suspended in M9 buffer and subsequently collected in a centrifuge tube. Then nematodes were washed multiple times with M9 buffer until the supernatant achieved near transparency and clarity. The washed nematodes of each group were individually dropped on a new NGM plate and air-dried at room temperature. Nematodes were allowed to crawl freely for a few minutes to remove the remaining bacterial cells from the body surface. Washed nematodes were gently transferred into the sterile water droplet for crawling, or a layer of M9 buffer for swimming on a 3 cm NGM plate. After acclimation period, a 2-min long video of nematode motion was recorded by using the SMZ745T dissecting microscope (Nikon, Tokyo, Japan) equipped with charge-coupled device camera. The locomotion performance of each group was analyzed using ImageJ software (National Institutes of Health, Bethesda, MD, USA), utilizing the wrMTrck plugin for detailed motion tracking and assessment (http://www.phage.dk/plugins/wrmtrck.html). One body bend was defined as complete bending of the worm body in one direction to the outermost angle and back to the initial posture.

#### 2.2.4 Phalloidin staining for muscle mass

Alexa Fluor™ 488 dye conjugates of phalloidin (A12379; Invitrogen, Thermo Fisher Scientific, Waltham, MA, USA) was used to stain actin filaments (F-actin) according to previously published methods (Kalichamy et al., 2020; Romani and Auwerx, 2021; Kim et al., 2023) with slight modifications. Briefly, the nematodes of each experimental groups were suspended from the plate with M9 buffer and subsequently collected in a 1.5 mL centrifuge tube. Nematodes were washed twice with M9 buffer and then snap frozen by immerging the centrifuge tube in liquid nitrogen. After completely removing residual moisture in the centrifuge tube, the washed nematodes were permeabilized using ice-cold acetone. Subsequently, nematodes were stained with Alexa Fluor™ 488 phalloidin overnight at room temperature in the dark. After washing twice with M9 buffer, the stained nematodes were mounted onto glass slides covered with a 2% agarose pad. Observation and image capture were conducted using the IX51 inverted microscope (Olympus, Tokyo, Japan) which emits a green fluorescent signal. Each nematode was traced using a polygon tool, and then the staining area ratio was calculated based on the highest peak of green fluorescence threshold in the ImageJ software.

#### 2.2.5 Oil-red O staining for body fat

Oil-red O (ORO) staining was performed following previously published methods (Nakagawa et al., 2016) with slight modifications. Briefly, the nematodes of each experimental groups were suspended from the plate with M9 buffer and subsequently collected in a 1.5 mL centrifuge tube. Nematodes were washed three times with M9 + 0.1% Triton X-100 and then permeabilized using M9 buffer containing 2% paraformaldehyde for 1 h. After washing away the paraformaldehyde with M9 buffer, the pellet of nematodes was treated with 60% isopropanol for 15 min. The ORO stock solution was diluted with water to 60% and the precipitate was removed with a 0.2 mm filter. The nematodes were stained overnight rocking with ORO solution at room temperature in the dark. After washing twice with M9 + 0.1% Triton X-100, the stained-nematodes were mounted on a glass slide with a 2% agarose pad. Observation and image capture were conducted using a dissecting microscope. Each nematode was traced using a polygon tool, and the staining area ratio and average intensity of staining were calculated based on the fixed threshold in the ImageJ software.

#### 2.2.6 Glycogen content analysis

For *in situ* glycogen storage analysis, iodine staining was performed as previously described (Gusarov et al., 2017). Briefly, the nematodes in each experimental groups were individually collected, washed with M9 buffer and transferred to fresh NGM plates. Following air-drying to eliminate moisture, the nematode-containing plates were inverted and placed on top of a 100 g bottle of iodine for 2 min. Observation and image capture were conducted using a dissecting microscope. The glycogen content of each nematode group was measured using a commercial assay kit, following previously published methods (Gusarov et al., 2017; Zecic et al., 2022; Seo et al., 2018) and the manufacturer's instructions. The nematodes in each experimental groups were individually collected and washed with ddH_2_O in a 1.5 mL centrifuge tube, and then put on ice. Samples were sonicated using the Qsonica Microson XL2000 Microprobe (Thermo Fisher Scientific) at 4 W for 30 s in 3 × 10 s bursts. Then the lysates were boiled for 10 min and centrifuged at 14000×*g* for 10 min at 4 °C. Supernatants were collected and total glycogen was measured using a Glycogen Assay Kit (MAK016; Sigma-Aldrich, St. Louis, MO, USA) and a microplate spectrophotometer. Glycogen levels were normalized to protein content measured with Bradford Assay Kit (Bio-Rad Laboratories, Hercules, CA, USA).

### 2.3 Animal study

#### 2.3.1 Animals and experimental design

Male 7-week-old ICR mice were purchased from BioLASCO (Charles River Licensee Co., Yi-Lan, Taiwan). All mice were housed under humidity-controlled (65 ± 5%) and temperature-controlled (24 ± 2 °C) conditions, maintained on a 12:12 light-dark cycle, and provided with Laboratory Rodent Diet 5001 feed (LabDiet, Columbia, MO, USA) and distilled water *ad libitum*. All animal experiments in this study were conducted in accordance with the guidelines of protocol 20220308-A002 and approved by the Institutional Animal Care and Use Committee of Providence University (Taichung, Taiwan). After a 2-week acclimation period, the mice were randomly divided into the following four groups, with 12 mice each: (1) Control (ddH_2_O, 10 mL/kg body weight [BW]); (2) TWK10-1 × (0.205 g/kg BW, corresponding to 1 × 10^10^ CFU in an adult human per day); (3) TWK10-2 × (0.410 g/kg BW, corresponding to 2 × 10^10^ CFU in an adult human per day); and (4) TWK10-5 × (1.025 g/kg BW, corresponding to 5 × 10^10^ CFU in an adult human per day). The mice were administered the test substances orally for six consecutive weeks. The conversion of mouse and human doses is based on the practical guidelines provided by the U.S. Food and Drug Administration (Shin et al., 2010; Nair and Jacob, 2016; Guidance for industry, 2005).

#### 2.3.2 Exercise performance

The evaluation of mouse exercise performance was performed following previously published methods (Chen et al., 2016). For the forelimb grip strength test, a low-force testing system (Model-RX-5; Aikoh Engineering, Nagoya, Japan) was used to measure the forelimb grip strength of treated mice. In brief, mice grasped the pull bar on the grip wire, which was gradually moved backward until they lost their grip. Grip strength was measured multiple times, and the longest duration for each trial was recorded. Endurance time was tested using a swim-to-exhaustion test. After 12 h of fasting, weight-bearing mice (5% of their body weight) were placed in a water tank. The mice were forced to swim until their heads remained submerged for 8 s without resurfacing, and the time was recorded.

#### 2.3.3 Glycogen content and body composition analysis

After the mice were euthanized, the liver, muscles (including quadriceps and gastrocnemius), and fat tissues (epididymal fat pad [EFP], inguinal white adipose tissue [iWAT], and retroperitoneal white adipose tissue [rpWAT]) were precisely excised and weighed. Portions of the muscle and liver tissues were stored in liquid nitrogen for glycogen content analysis. The samples were homogenized in five volumes (w/v) of tissue homogenate solution using the Bullet Blender (Next Advance, Inc., Cambridge, MA, USA). After centrifugation, the supernatant was collected and then incubated in an iodine (I_2_-KI) solution containing saturated CaCl_2_ (Chamberland and Rioux, 2010). The glycogen content was quantified using a standard curve prepared with commercial glycogen (Sigma-Aldrich), allowing the calculation of glycogen storage changes in liver and muscle tissues.

### 2.4 Clinical study

#### 2.4.1 Clinical trial experimental design

A total of 30 healthy male adults (aged 20–40 years old) without professional athletic training were recruited for this double-blind, placebo-controlled trial. Subjects were excluded from this study if they had smoking or drinking habits; or had any known disorders, including heart/cardiopulmonary disease, diabetes, neuromuscular disorder, neurological disease, autoimmune disease, peptic ulcers, ulcerative colitis, or other chronic diseases. All subjects were asked to maintain their usual diet and lifestyle, and prohibited from consuming any other nutritional supplements, including probiotics, prebiotics, fermented products (yogurt or other foods), or antibiotics, to avoid interference during supplementation. The subjects were divided into the following two groups based on their maximal rate of oxygen consumption (VO_2max_, mL/kg/min): control group (*n* = 15) and TWK10 group (1 × 10^10^ CFU/day, *n* = 15). After a 2-week washout period, the subjects were required to take two placebo [mixture of maltodextrin (99%) and microcrystalline cellulose (1%)] or TWK10 capsules daily after meals for 6 weeks, after which exercise endurance performance, grip strength, fatigue-related parameters, and body composition were assessed. This study was conducted in accordance with the Declaration of Helsinki and all procedures were reviewed and approved by the Institutional Review Board of Landseed International Hospital (LSHIRB No. 19-027-A2; Taoyuan, Taiwan). All subjects provided written informed consent.

#### 2.4.2 Exercise performance

The evaluation of human exercise performance was performed following previously published methods (Lee et al., 2021b). Handgrip strength was measured in kilograms using the Takei digital grip strength meter (T.K.K.5401; Takei Scientific Instruments Co., Ltd, Niigata, Japan). During the test, subjects were asked to squeeze the gripper with one hand with maximum effort, maintain the squeeze for at least 5 s, and repeat the test with the other hand in 60-s intervals to prevent fatigue. The maximum grip strength of three consecutive measurements was recorded for both the left and right hands. The subjects performed a maximum endurance test using a treadmill. They warmed up at 60% VO_2max_ intensity for 5 min and then started the endurance running test at an 85% VO_2max_ workload. To assess maximum exercise tolerance, their physical conditions were monitored every 5 min using heart rate and Borg's rating of perceived exertion until subjects experienced exhaustion. The sustained exercise duration was recorded as the endurance index.

#### 2.4.3 Fatigue-associated biochemical indices and body composition analysis

To assess fatigue-related indicators, subjects fasted for 8 h before the fixed intensity exercise challenge at 60% VO_2max_. Blood samples were collected via an arm venous catheter at baseline (0), 15 min (E15), and 30 min (E30) during the exercise challenge, and 20 min (R20) and 60 min (R60) during recovery. Serum lactate and ammonia levels were measured using an automatic analyzer (7060; Hitachi, Chiyoda, Tokyo). After an 8-h fast, body composition was measured using the InBody 570 body composition analyzer (Biospace, Inc., Seoul, Korea), which uses bioelectrical impedance analysis (BIA) technology. This device uses a multi-frequency principle, screening at 1, 5, 50, 260, 500, and 1000 kHz in 60 s. Subjects cleaned their hands and feet before standing on the electrodes and holding the sensing handles with both hands, keeping their arms open at a 30° angle without speaking or moving during the measurement.

### 2.5 Statistical analyses

Data are expressed as the mean ± standard deviation. Statistical analyses were performed using GraphPad Prism 7.04 (GraphPad Software, San Diego, CA, USA). One-way analysis of variance (ANOVA) with Tukey's *post-hoc* test was used for multiple group comparisons. The Kruskal–Wallis test with Dunn's *post-hoc* test was used for multiple non-parametric comparisons. Within-group differences (before and after timeline) were analyzed using the paired Student's *t*-test. In the clinical study, differences between groups (control vs. TWK10 groups) were analyzed using the unpaired Student's *t*-test for parametric comparisons and the Mann–Whitney *U* test for non-parametric comparisons, including changes in muscle weight and body fat.

## 3 Results

### 3.1 *C. elegans* study

#### 3.1.1 Impact of LAB strains on the lifespan of *C. elegans*

**Fig. 1:**
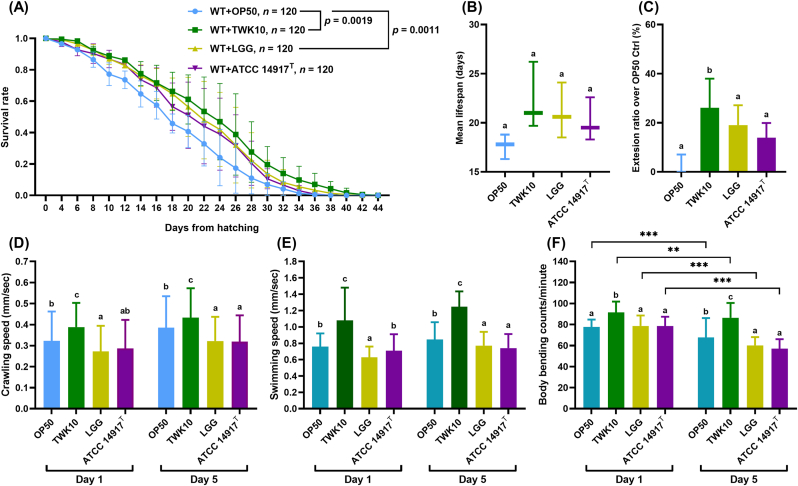
Lifespan and locomotion in *C. elegans*. (A) Lifespan assay of LAB-treated *C. elegans*. The survival rates of each group are presented as the mean ± SD, and the statistical differences between the four groups were analyzed using the log–rank (Mantel–Cox) test; each of the three independent experiments used 120 *C. elegans* (20 nematodes/well). WT, *C. elegans* wild-type N2 strain (B) MLS and (C) MLS extension rate of LAB-treated *C. elegans* from three independent experiments. Data are presented as the mean ± SD and were analyzed by the Kruskal–Wallis test with Dunn *post-hoc* test. Locomotion behavior analysis of LAB-treated *C. elegans* includes (D) crawling speed, (E) swimming speed, and (F) body bending frequency during swimming. Data are presented as the mean ± SD and were analyzed using one-way ANOVA with Tukey's *post-hoc* test; *n* ≥ 100. Different letters (a, b, c) indicate a significant difference at *p* < 0.05. Significant differences in the body bending frequency between Day 1 and Day 5 in each group were analyzed using the paired *t*-test and indicated by ∗∗*p* < 0.01, ∗∗∗*p* < 0.001. OP50, control group received only *E. coli* OP50; LGG, *L. rhamnosus* GG; ATCC 14917 ^T^, *L. plantarum* ATCC 14917^T^.

In comparison to the OP50 group, the TWK10 group demonstrated the most significant enhancement in daily survival rate and lifespan extension followed by the LGG group (*p* < 0.01 and *p* < 0.05, respectively; Fig. 1A). The ATCC 14917^T^ group demonstrated the least impact, with no statistically significant difference compared to the OP50 group. Regarding the MLS calculated based on the survival rate per survival day, the TWK10 group exhibited the greatest MLS of 22.3 ± 3.4 days, followed by the LGG group with an MLS of 21.1 ± 2.8 days and the ATCC 14917^T^ group with an MLS of 20.1 ± 2.3 days, all exceeding that of the OP50 group (17.6 ± 1.2 days). However, no significant differences were observed between the four groups (Fig. 1B). A comparison of the ratio of the MLS of each group to that of the OP50 group in the same batch revealed a significant increase in the TWK10 group with an MLS extension of 26.1 ± 11.9 % (*p* < 0.05, Fig. 1C).These results demonstrate that TWK10 significantly prolongs the lifespan of *C. elegans* compared to LGG and ATCC 14917^T^.

#### 3.1.2 TWK10 significantly enhances locomotion in adult *C. elegans*

The exercise performance of LAB-fed nematodes was assessed by analyzing the locomotor behavior including crawling and swimming. In the young and mature adult stages (Days 1 and 5, respectively), the TWK10 group demonstrated a significantly higher (*p* < 0.01 and *p* < 0.05, respectively) crawling speed compared to the OP50 group. However, the LGG and ATCC 14917^T^ groups demonstrated no significant improvement in crawling speed compared to the OP50 group (Fig. 1D). A similar trend was observed in swimming behavior, as the TWK10 group exhibited a higher swimming speed compared to the OP50 group on Days 1 and 5 (both *p* < 0.001; Fig. 1E). The TWK10 group also demonstrated a significantly elevated body bending frequency compared to the OP50 group on Days 1 and 5 (both *p* < 0.001; Fig. 1F), whereas the LGG and ATCC 14917^T^ groups demonstrated no significantly improvement in body bending frequency compared to the OP50 group Notably, we observed a significant age-related decline in body bending frequency, as previously reported (Glenn et al., 2004), in the OP50, LGG and ATCC 14917^T^ groups (Day 1 vs. Day 5; all *p* < 0.001), while a relatively smaller decline observed in the TWK10 group (Day 1 vs. Day 5; *p* < 0.01). These results demonstrate that TWK10 has a significant and sustained effect on locomotion promotion in *C. elegans*.

#### 3.1.3 TWK10 significantly enhances muscle mass in adult *C. elegans*

**Fig. 2:**
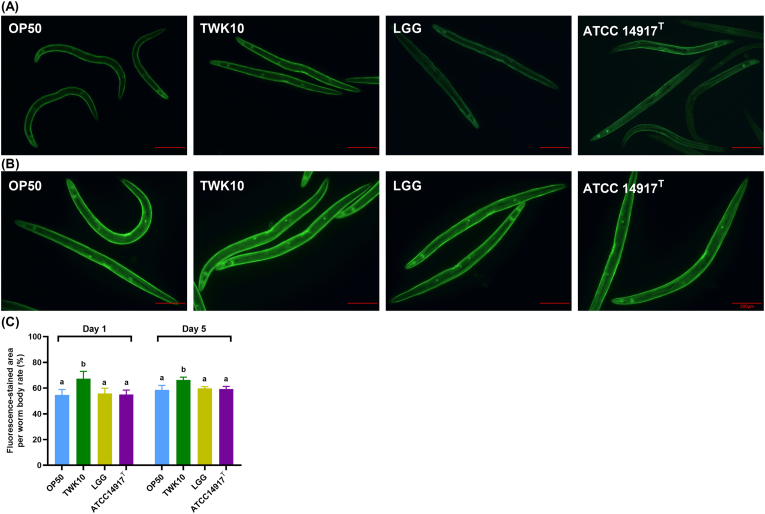
Muscle mass in *C. elegans*. Phalloidin staining visualizes *in situ* filamentous actin-positive regions in LAB-treated *C. elegans* on (A) Day 1 and (B) Day 5. (C) Quantification of filamentous actin-positive area in single nematodes; *n* = 30. Data are presented as the mean ± SD and were analyzed by the Kruskal–Wallis test with Dunn's *post-hoc* test. Different letters (a, b, c) indicate a significant difference at *p* < 0.05. Scale bar = 200 μm. OP50, control group received only *E. coli* OP50; LGG, *L. rhamnosus* GG; ATCC 14917 ^T^, *L. plantarum* ATCC 14917^T^.

To assess whether LAB feeding affects the muscle mass of *C. elegans*, we visualized and quantified muscle fiber areas in individual nematodes using actin filaments (F-actin)-specific staining. The morphology of *C. elegans* muscle tissue, including body wall, pharyngeal and vulval muscles, was successfully visualized by fluorescence imaging (Fig. 2A and B). The area with fluorescent staining in the TWK10 group was significantly higher than those of OP50, LGG and ATCC 14917^T^ groups in Day 1 young adult (all *p* < 0.001; Fig. 2A and C). In Day 5 mature adults with larger body size, TWK10 group continued to exhibit significantly greater muscle mass than the other groups (all *p* < 0.001; Fig. 2B and C). These results indicate that TWK10 has a significant and sustained effect on improving muscle mass in *C. elegans*.

#### 3.1.4 Both TWK10 and LGG significantly reduced lipid accumulation in adult *C. elegans*

**Fig. 3:**
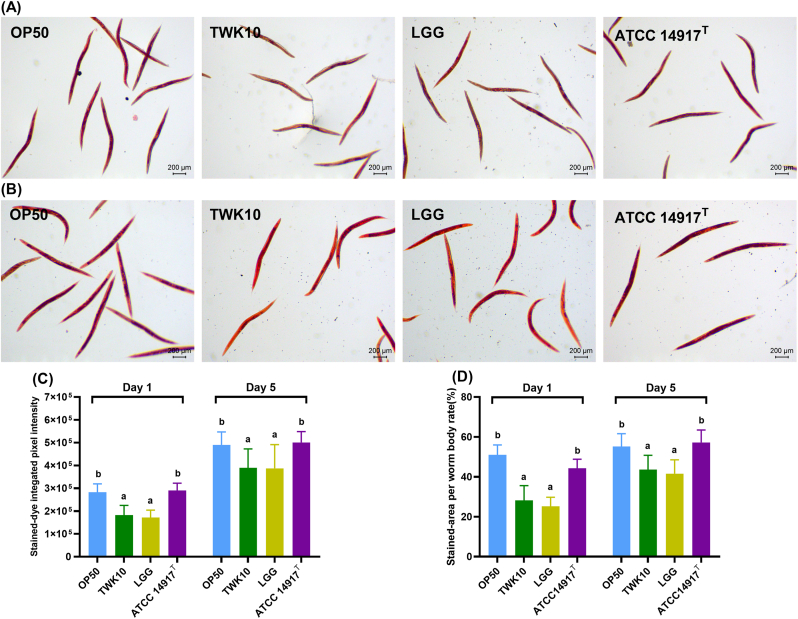
Lipid accumulation in *C. elegans*. ORO staining visualizes *in situ* lipid droplets accumulation in LAB-treated *C. elegans* on (A) Day 1 and (B) Day 5. (C) Quantification of ORO staining intensity and (D) stained area ratio of single nematodes. Data are expressed as the mean ± SD and were analyzed by the one-way ANOVA with Tukey's *post-hoc* test for parametric comparisons and Kruskal–Wallis test with Dunn's *post-hoc* test for non-parametric multiple comparisons test; *n* = 30. Different letters (a, b, c) indicate a significant difference at *p* < 0.05. Scale bar = 200 μm. OP50, control group received only *E. coli* OP50; LGG, *L. rhamnosus* GG; ATCC 14917 ^T^, *L. plantarum* ATCC 14917^T^.

We used ORO staining to evaluate the effects of LAB feeding on fat metabolism in *C. elegans*. ORO staining detected substantial amounts of both epidermal and intestinal lipid droplets in individual nematodes. The accumulation of fat in mature adults (Day 5) was higher than that in young adults (Day 1) in all experimental groups, with a notable increase in epidermal fat (Fig. 3A and B). The intensity of the ORO dye and the stained area per individual body were significantly reduced in both the TWK10 and LGG groups on the Days 1 and 5 (both *p* < 0.001), whereas those in the ATCC 14917^T^ group were not significantly different from the OP50 group on the Days 1 and 5 (Figs. A–D). These results indicate that both TWK10 and LGG have a significant and sustained effect on reducing body fat in *C. elegans*.

#### 3.1.5 TWK10 sustained increases glycogen storage in adult *C. elegans*

**Fig. 4:**
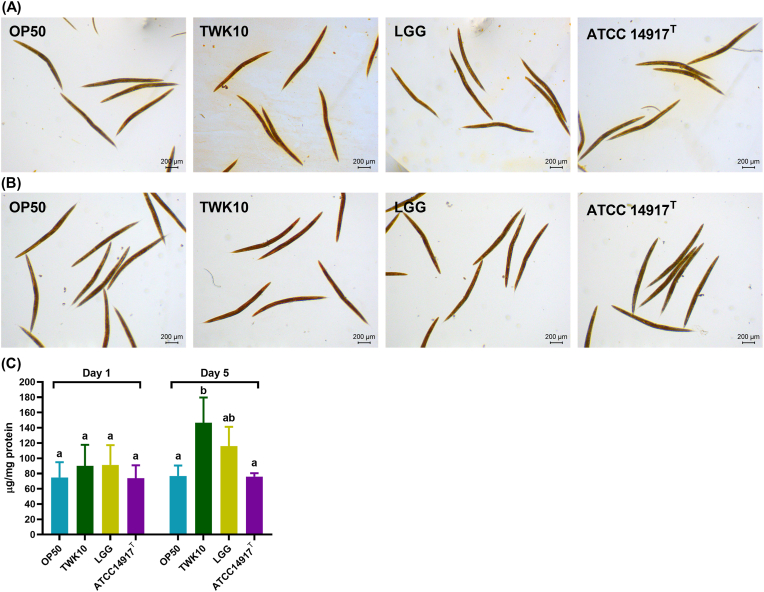
Glycogen storage levels in *C. elegans*. Iodine staining visualizes *in situ* glycogen storage regions in LAB-treated *C. elegans* on (A) Day 1 and (B) Day 5. (C) Glycogen content in nematodes from three independent experiments. Data are expressed as the mean ± SD and were analyzed by the one-way ANOVA with Tukey's *post-hoc* test. Different letters (a, b) indicate a significant difference at *p* < 0.05. Scale bar = 200 μm. OP50, control group received only *E. coli* OP50; LGG, *L. rhamnosus* GG; ATCC 14917 ^T^, *L. plantarum* ATCC 14917^T^.

We assessed the impact of LAB feeding on glycogen storage in *C. elegans* by using the iodine staining to visualize the *in situ* glycogen stores in individual nematodes, primarily in the pharynx, proximal oocytes, embryos in utero, intestine and tail hypodermis on Days 1 and 5. The TWK10 group exhibited the highest staining intensity, particularly in the intestine and oocytes regions, followed by the LGG group on both Day 1 and Day 5 (Fig. 4A and B). Subsequently, the total glycogen level in nematode cell lysates was determined with an enzymatic assay. On Day 1, the glycogen levels were elevated in the TWK10 and LGG groups compared to the OP50 and ATCC 14917^T^ groups, although the differences were not statistically significant. On Day 5, a significant increase was only observed in the TWK10 group in comparison to the ATCC 14917^T^ and OP50 groups (both *p* < 0.05). Glycogen storage in the LGG group on Day 5 showed no significant difference compared to that in the OP50 group (*p* = 0.2109; Fig. 4C). These results indicate that TWK10 has a sustained effect on elevating glycogen storage level in *C. elegans*.

#### 3.1.6 Batch efficacy verification of mass-produced TWK10 bacterial powder in *C. elegans*

**Fig. 5:**
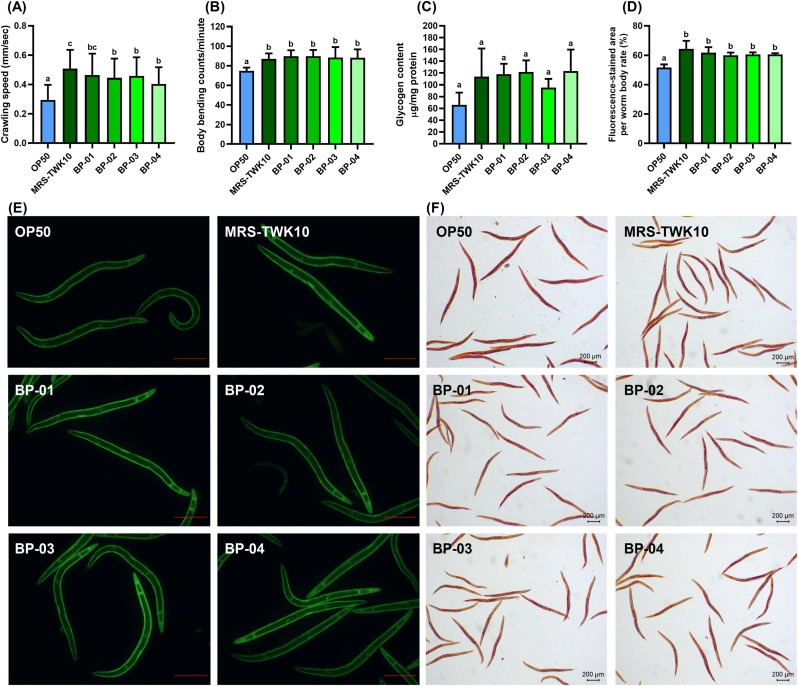
Equivalence assessment of the ergogenic efficacy of lab-prepared TWK10 and mass-produced TWK10 bacteri**al powder.** (A) Locomotion behavior analysis of crawling speed and (B) body bending frequency; *n* ≥ 100. (C) Glycogen content in nematodes from three independent experiments. (D) Quantification of (E) filamentous actin-positive area of single nematodes; *n* = 30. (F) ORO staining visualizes lipid accumulation in nematodes. Data are presented as the mean ± SD and were analyzed by the one-way ANOVA with Tukey's *post-hoc* test for parametric comparisons and Kruskal–Wallis test with Dunn's *post-hoc* test for non-parametric comparison. Different letters (a, b, c) indicate a significant difference at *p* < 0.05. Scale bar = 200 μm. OP50, control group received only *E. coli* OP50; BP, factory-produced bacterial powder; MRS, TWK10 bacterial cells using MRS broth.

Four batches of the mass-produced TWK10 bacterial bulk powder (BP-TWK10) and the laboratory-prepared TWK10 (MRS-TWK10) were adjusted to the same bacterial amount and subsequently assessed for equivalence in effects on *C. elegans*. The crawling speed of all BP-TWK10 groups was significantly increased (all *p* < 0.001) compared to the OP50 group, consistent with the results observed in the MRS-TWK10 group (Fig. 5A). No significant difference was observed in body bending frequency between the MRS-TWK10 group and the four batches of BP-TWK10 groups. However, all of these groups demonstrated a significant increase (all *p* < 0.001) compared to the OP50 group (Fig. 5B). Additionally, all BP-TWK10 groups showed the same elevation in glycogen storage as the MRS-TWK10 group (Fig. 5C). Phalloidin staining demonstrated a significant increase (all *p* < 0.001) in muscle mass in all four BP-TWK10 groups and the MRS-TWK10 group compared to the OP50 group (Fig. 5D and E). ORO staining showed that lipid accumulation was also decreased in these groups (Fig. 5F). Although differences in crawling speed between three BP-TWK10 groups and the MRS-TWK10 groups reached statistical significance, overall, most of the ergogenic indices showed no significant difference in degree among these five groups. Thus, verification based on locomotion, muscle mass, body fat mass, and glycogen content in *C. elegans* confirmed that the mass-produced TWK10 bacterial powder demonstrate ergogenic benefits equivalent to those of laboratory-prepared TWK10 cells.

### 3.2 Animal study

#### 3.2.1 Effects of mass-produced TWK10 on forelimb grip strength and swimming endurance in mice

**Fig. 6:**
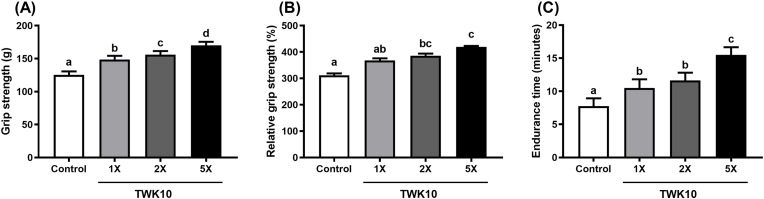
Effects of mass-produced TWK10 bacterial powder in mice on forelimb grip strength and swimming pe**rformance.** (A) Total and (B) relative forelimb grip strength of mice. (C) Swimming endurance time of mice; *n* = 12. Data are expressed as the mean ± SD. Statistical differences among groups were analyzed by the one-way ANOVA with Tukey's *post-hoc* test. Non-parametric data were statistically analyzed by the Kruskal–Wallis test with Dunn's *post-hoc* test. Different letters (a, b, c, d) indicate a significant difference at *p* < 0.05.

The mass-produced TWK10 was administered to mice at doses of TWK10-1 × , TWK10-2 × , and TWK10-5 × . After 6 weeks of administration, all TWK10 groups showed significant improvement in grip strength and swimming endurance compared to the control group (Fig. 6A–C). The TWK10-5× group showed the highest grip strength (170 ± 5 g), followed by the TWK10-2× group (156 ± 6 g) and the TWK10-1× group (148 ± 6 g). The differences in grip strength among these three dosage groups were statistically significant (all *p* < 0.001, except *p* < 0.01 between TWK10-1 × and TWK10-2 × ; Fig. 6 A). After normalization to individual body weight, the relative grip strength of the TWK10-5 × and TWK10-2× group was still significantly different compared to the control group (both *p* < 0.001; Fig. 6B). The difference in relative grip strength between the TWK10-1 × and TWK10-5× groups was statistically significant (*p* < 0.001; Fig. 6B). The swimming endurance time in the TWK-5× group was also significantly higher than that in the TWK10-2 × and TWK10-1× groups (both *p* < 0.001). Although the TWK10-2× group showed higher endurance time than the TWK10-1× group, the difference was not statistically significant (Fig. 6C). These results demonstrate that mass-produced TWK10 significantly improves mouse exercise performance in a dose-dependent manner.

#### 3.2.2 Effect of mass-produced TWK10 on muscle mass and glycogen storage in the muscle and liver of mice

After the mice were sacrificed, the livers, muscles, and adipose tissues of each group were collected for analysis of TWK10 efficacy indicators. The liver glycogen levels were significantly elevated in the TWK10-1 × , TWK10-2 × and TWK10-5× groups, with fold changes of 1.37, 1.57 and 2.17, respectively, compared to the control group (all *p* < 0.001). The differences in liver glycogen levels among these three dosage groups were statistically significant (all *p* < 0.001). Similarly, intramuscular glycogen levels were significantly elevated in these three TWK10 groups, with fold changes of 1.63, 1.75, and 2.20, respectively, compared to the control group (all *p* < 0.001). Intramuscular glycogen level was also significantly higher in the TWK-5× group than in the TWK10-2 × and TWK10-1× groups (both *p* < 0.001, Table 1). Although the TWK10-2× group showed a higher intramuscular glycogen than the TWK10-1× group, the difference was not statistically significant. The relative tissue weight (adjusted for individual body weight percentage) of quadriceps was increased in all TWK10 groups compared to the control group, with statistical significance in the TWK10-2 × and TWK10-5× groups (both *p* < 0.01). Furthermore, a significant elevation in relative gastrocnemius weight was also observed in all TWK10 groups compared to that of the control group (all *p* < 0.01; Table 1). With respect to adipose tissues, the relative weights of EFP, iWAT, and rpWAT were slightly decreased in all TWK10 groups compared to the control group, though these differences were not statistically significant (Table 1). These results demonstrate that TWK10 increases muscle mass and reduces body fat, leading to a significantly improves body composition in mice. The efficacy of TWK10 is dependent on the administered dose, with increased glycogen storage observed in a dose-dependent manner.

**Table 1:** Effects of mass-produced TWK10 bacterial powder on the glycogen content, muscle weight, and body fat of mice.

	Control	TWK10-1 ×	TWK10-2 ×	TWK10-5 ×
Liver glycogen (mg/g)	10.75 ± 1.37 ^a^	14.75 ± 1.12 ^b^	16.98 ± 1.28 ^c^	23.34 ± 1.46 ^d^
Muscle glycogen (mg/g)	0.87 ± 0.06 ^a^	1.42 ± 0.22 ^b^	1.52 ± 0.24 ^b^	1.91 ± 0.18 ^c^
Relative quadriceps weight (%)	1.31 ± 0.03 ^a^	1.35 ± 0.07 ^ab^	1.37 ± 0.04 ^b^	1.36 ± 0.04 ^b^
Relative gastrocnemius weight (%)	0.90 ± 0.02 ^a^	1.01 ± 0.02 ^b^	1.00 ± 0.03 ^b^	1.05 ± 0.04 ^b^
Relative EFP weight (%)	1.20 ± 0.09 ^a^	1.11 ± 0.09 ^a^	1.12 ± 0.09 ^a^	1.10 ± 0.15 ^a^
Relative iWAT weight (%)	0.86 ± 0.06 ^a^	0.84 ± 0.08 ^a^	0.83 ± 0.06 ^a^	0.82 ± 0.04 ^a^
Relative rpWAT weight (%)	0.38 ± 0.05 ^a^	0.36 ± 0.06 ^a^	0.37 ± 0.07 ^a^	0.37 ± 0.07 ^a^

Data are expressed as the mean ± SD and were analyzed by the one-way ANOVA with Tukey's *post-hoc* test (parametric) or the Kruskal–Wallis test with Dunn's *post-hoc* test (non-parametric). Different letters (a, b, c, d) indicate a significant difference at *p* < 0.05. EFP, epididymal fat pad; iWAT, inguinal white adipose tissue; rpWAT, retroperitoneal white adipose tissue.

### 3.3 Clinical study

#### 3.3.1 Effect of mass-produced TWK10 on handgrip strength and endurance performance in healthy male adults

**Fig. 7:**
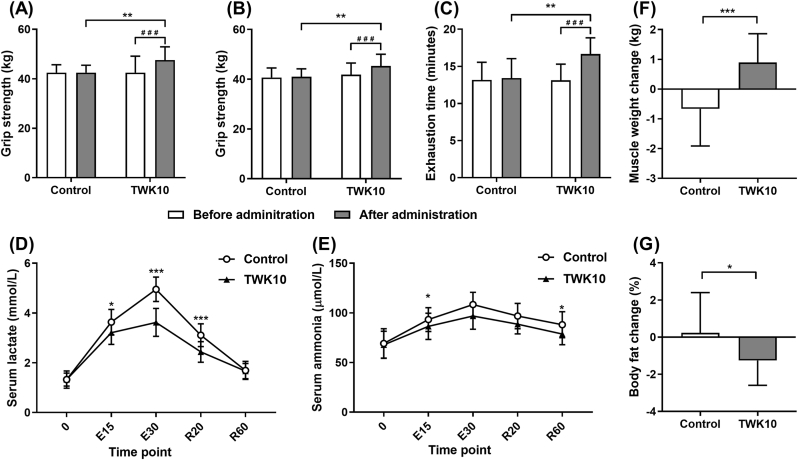
Ergogenic effects of mass-produced TWK10 bacterial powder in healt**hy adults.** (A) Exercise performance evaluation, including grip strength of (A) right hand and (B) left hand (C) exhaustion time. (D) Serum lactate and (E) ammonia levels during exercise and rest periods after 6 weeks of administration. (F) Muscle mass and (G) body fat changes in body composition of individual subjects. Data are expressed as the mean ± SD. Statistical significance between groups was analyzed using the unpaired *t*-test for parametric comparison, and the Mann–Whitney *U* test for non-parametric comparisons. Significance is shown by: ∗*p* < 0.05, ∗∗*p* < 0.01, ∗∗∗*p* < 0.001. Intragroup differences were analyzed using the paired *t*-test and indicated by ^###^*p* < 0.001.

The demographic profiles, characteristics and baseline values of the two groups were comparable prior to treatment, with no significant differences observed (Table 2 and Fig. 7). After 6 weeks of administration, no significant difference was observed in grip strength compared to the baseline values in the control group. By contrast, the grip strength in the TWK10 group demonstrated a 1.12-fold (*p* < 0.001; Fig. 7A) and a 1.08-fold (*p* < 0.001; Fig. 7B) increase in right- and left-hand grip strength, respectively, compared to their baseline values. Grip strength in the TWK10 group after administration was significantly increased by 1.12-fold (*p* < 0.01; Figs. 7A) and 1.1-fold (*p* < 0.01; Fig. 7B) in the right- and left-hand grip strength, respectively, compared to the control group. The endurance performance of the subjects was assessed using the time-to-exhaustion test with 85% VO_2max_ workload. Similarly, the TWK10 group exhibited a significant 1.24-fold increase in exhaustion time compared to the control group (*p* < 0.01; Fig. 7C) after a 6-week administration period. Furthermore, the TWK10 group demonstrated a significant 1.27-fold (*p* < 0.001) increase in exhaustion time after administration compared to their baseline values, whereas no significant change was observed in the control group. These results demonstrate that administration of 1 × 10^10^ CFU mass-produced TWK10 significantly improves the exercise performance and functional capacity of human subjects.

**Table 2:** General characteristics of the subjects.

	Control	TWK10
**Subjects**	*n =* 15	*n =* 15
**Height (cm)**	172.5 ± 5.3	173.5 ± 6.0
**Weight (kg)**	69.3 ± 7.7	70.6 ± 12.1
**Age (y)**	20.5 ± 1.5	20.2 ± 1.4
**VO**_**2max**_**(mL/kg/min)**	53.5 ± 8.1	53.1 ± 6.3

Subjects were randomly assigned to the control and TWK10 groups based on VO_2max_ measurements before treatment. Data are expressed as the mean ± SD and were analyzed by the Student's unpaired *t*-test.

#### 3.3.2 Effect of mass-produced TWK10 on physiological adaptation and body composition in healthy male adults

Physiological adaptation in subjects was assessed using fatigue-related indices based on the fixed-intensity exercise challenge with 60% VO_2max_ workload. Following a 6-week administration period, the TWK10 group demonstrated a significant reduction in circulating lactate levels at E15, E30 and R20 compared to the control group (*p* < 0.05, *p* < 0.001, and *p* < 0.001, respectively; Fig. 7D). Furthermore, the circulating ammonia levels in the TWK10 group were significantly reduced at E15 and R60 compared to the control group (both *p* < 0.05; Fig. 7E). As for the body composition, the subjects in the TWK10 group showed an average increase in muscle mass gain of 0.9 kg, whereas the subjects in the control group showed an average reduction in muscle mass of 0.7 kg (*p* < 0.001; Fig. 7F). Furthermore, subjects in the TWK10 group showed an average reduction in body fat of 1.26%, whereas subjects in the control group showed an average increase in body fat of 0.23% (*p* < 0.05; Fig. 7G). These results demonstrate that mass-produced TWK10 significantly improves physiological adaptation and body composition in human subjects.

## 4 Discussion

The combination of *C. elegans* with other *in vitro* methods to validate or screen probiotics and related substances has become a widely adopted strategy (Zanni et al., 2017; Guantario et al., 2018). Based on the extensive experience with *C. elegans* in aging research, the effect of probiotic supplementation on nematode lifespan is typically considered, followed by further analysis of oxidative stress and immunosenescence pathways (Roselli et al., 2019). Our results revealed that TWK10 significantly enhanced the daily survival rate and lifespan of *C elegans*, outperforming both LGG and ATCC 14917^T^ (Fig. 1A–C). LGG, as a world-renowned immunomodulatory strain, has been proven to enhance *C. elegans* lifespan and resistance to food-borne pathogen infection (Yun et al., 2021), while the slight lifespan increase with ATCC 14917^T^ may be closely related to its immunomodulatory properties, as demonstrated in studies on LGG and other strains (Lee et al., 2011; Capurso, 2019; Yun et al., 2021; Roselli et al., 2019; Hassan et al., 2020). The improvements of TWK10 in aging-related muscle, metabolism, and cognitive deficits of senescent organisms indicated its influence on anti-aging pathways of the host (Lee et al., 2021a, 2021b). The above findings demonstrate that *C. elegans*, with its reproducible results, can provide reliable insights for both screening and beneficial identification of microorganism.

Improvements in exercise performance, muscle mass, glycogen and corresponding energy utilization, and reductions in body fat have been consistently observed in most TWK10 animal and clinical studies (Chen et al., 2016, 2020; Huang et al., 2018, 2019; Lee et al., 2021a, 2021b, 2022). In our *C. elegans* study, TWK10 also increased locomotion and muscle mass (Fig. 1, Fig. 2), along with reducing fat mass and increasing glycogen storage (Fig. 3, Fig. 4). By contrast, ATCC14917^T^, a type strain of *L. plantarum* subsp. *plantarum*, was significantly inferior to TWK10 in lifespan and ergogenic effects (Fig. 1, Fig. 2, Fig. 3, Fig. 4), despite their similarities in origin and genetic content (Hsu et al., 2022). This discrepancy suggests that the 228 strain-specific genes in TWK10, which differ from those in ATCC14917^T^, may provide insights into its unique efficacy mechanisms (Hsu et al., 2022). The LGG trials reported the immunoregulatory effects on alleviating similar symptoms but did not mention any improvements related to exercise (Kekkonen et al., 2007; Moreira et al., 2007). At present, there are no studies indicating that LGG or ATCC14917^T^ enhance the exercise capacity or skeletal muscle mass of the host organism. Meanwhile, TWK10, a probiotic with well-documented ergogenic effects (Chen et al., 2016, 2020; Huang et al., 2018, 2019; Lee et al., 2021a, 2021b, 2022), has consistently demonstrated enhancements in locomotion and muscle mass in *C. elegans* (Fig. 1, Fig. 2). Given the conserved muscle cell structure and sarcomere function between *C. elegans* and vertebrates (Gieseler et al., 2017; Fukushige et al., 2006), our results indicate that the *C. elegans* model is sufficient to distinguish strain-specific ergogenic benefits and can be used as a preliminary screening and validation for such applications (Gjorgjieva et al., 2014; Ali et al., 2023).

The glycogen and fat metabolism of nematodes is connected by the tricarboxylic acid cycle, similar to that of mammals, reflecting carbohydrate energy storage and utilization (Watts and Ristow, 2017). Both TWK10 and LGG had similar effects on reducing body fat in *C. elegans* (Fig. 3). Yun et al. (2021) reported that LGG upregulates mitogen-activated protein kinase pathways by promoting PMK-1 activity in *C. elegans*, which is highly associated with AMP-activated protein kinase (AMPK) signaling (Ju et al., 2022). Since chronic AMPK activation has been shown to lead to glycogen accumulation in multiple model systems as well as in *C. elegans* (Possik et al., 2015; Possik and Pause, 2016), we propose that future investigations be conducted to elucidate the relationship between TWK10 administration and AMPK activity. Sustained glycogen accumulation was also observed in the insulin-like growth factor-1 (IGF-1) receptor ortholog abnormal dauer formation protein 2 (daf-2) mutant *C. elegans* (Watts and Ristow, 2017; Gusarov et al., 2017; Zecic et al., 2022). Many previous studies have reported the longevity of daf-2 mutants (Bulger et al., 2017; Kimura et al., 1997), which further supports the benefits of insulin regulation for longevity (Li et al., 2021; Templeman et al., 2017). Interestingly, *daf-2* mutant shows an increased presence of muscle cell structural components (Depuydt et al., 2013), while DAF-2 depletion in middle-aged nematode muscle improves motility (Roy et al., 2022). Considering the results of TWK10 extending lifespan (Fig. 1A–C), the analogy between TWK10 administration and *daf-2* downregulation suggests the possibility of TWK10 modulation on insulin/IGF-1 signaling in *C. elegans*. Improvements in body compositions in mammals receiving TWK10 may also echo the functions of IGF-1 and enhance insulin sensitivity (Friedrich et al., 2012; Ahmad et al., 2020). Previous proteomic studies showed that TWK10 upregulates host liver peroxisomal β-oxidation, fatty acid transport and the endoplasmic reticulum stress response (Chen et al., 2020), suggesting an insulin sensitivity-enhanced liver that functions as an energy source for peripheral organs (Shannon et al., 2021; Yao et al., 2023; Lai et al., 2023). Intestinal butyrate and its producing bacteria, which were consistently enhanced in mice and humans administered TWK10 (Lee et al., 2021a, 2022; Chen et al., 2020), also contribute to insulin sensitivity enhancement (Cui et al., 2022; Gao et al., 2009). However, our *C. elegans* results demonstrate significant benefits from TWK10 despite the absence of a distinct liver organ and butyrate-producing bacteria (Fig. 1, Fig. 2, Fig. 3, Fig. 4, Fig. 5). Therefore, we speculate that TWK10 may enhance host insulin sensitivity, including in *C. elegans,* through direct and indirect pathways within multi-organ axis, thereby achieving its ergogenic and longevous benefits (Giron et al., 2022; Yoshida and Delafontaine, 2020). Given the complexity of insulin/IGF-1 expression, which is influenced by multiple hormones and nutritional status in mammals, *C. elegans* could be a highly efficient approach for tracking the action of TWK10 (Bulger et al., 2017; Depuydt et al., 2014).

The probiotic field has long called for confirmation that industrial production of probiotic strains must retain the functional health properties for which they were originally selected (Ahire et al., 2023; Tuomola et al., 2001). In the past, the assessments often relied on the characteristics of tolerance to environmental stress, cytokine response from immune cell, and adhesion to intestinal epithelial cells (Torres-Maravilla et al., 2022; Tuomola et al., 2001), but these methods cannot adequately represent the full efficacy of novel strains like TWK10, which influence multiple pathways in the host. Using the *C. elegans* model, we confirmed the equivalence of laboratory-prepared and mass-produced TWK10 in ergogenic efficacy (Fig. 5), indicating that the industrial-manufacturing and additives, including cryoprotectants and excipients, neither enhance nor inhibit the effectiveness of TWK10. In mouse, we observed a dose-dependent effect on liver and muscle glycogen content and exercise performance, but not on muscle mass increase (Fig. 6 and Table 1), which consistent with previous strain studies (Chen et al., 2016; Huang et al., 2019; Lee et al., 2021b). The enhancements in exercise performance observed with the oral administration of 1 × 10^10^ CFU/day mass-produced TWK10 indicated the glycogen-enhancing benefits, accompanied by a reduction in fatigue-related indices in humans (Fig. 7A–E). In short, improvements in athleticism, body composition, and the utilization of carbohydrate energy reserves were consistently observed in *C. elegans*, mice, and human subjects following the administration of mass-produced TWK10 (Fig. 5, Fig. 6, Fig. 7 and Table 1). Thus, our comprehensive analysis confirmed both the efficacy equivalence and the dose-manipulation stability of mass-produced TWK10. On the premise of obtaining equivalent efficacy information, *C. elegans* indeed can serves as a rapid, simplified, and reliable alternative analysis model for those performed in mice and humans.

The viability and stability of probiotics during manufacturing, storage, and transportation also remain a significant concern, as several environmental factors compromise bacterial life performance, thereby diminishing the intended health benefits (Bianchi et al., 2020; Fenster et al., 2019). Accurate and rigorous clinical research on probiotics is indispensable, but conducting such studies solely for general industrial purposes can be challenging and inefficient. An implementation method that balances efficiency, representativeness, and rationality would significantly advance the progress in microbial industry. In this study, we confirmed that the ergogenic effects of TWK10 were consistent in *C. elegans* (Fig. 1, Fig. 2, Fig. 3, Fig. 4) and fully verified the efficacy stability of mass-produced TWK10 through comprehensive analysis, providing a basis for the *C. elegans* approach in TWK10-related industrial applications (Fig. 5, Fig. 6, Fig. 7 and Table 1). Moreover, *C. elegans* offers practical advantages in operational capabilities for tracking biomarkers under various conditions (Kumar et al., 2020; Ortiz de Ora and Bess, 2021; Poupet et al., 2020; Kim et al., 2023; Seo et al., 2018; Gu et al., 2022). We believe that *C. elegans*, as a versatile and efficient model organism, holds significant potential for ergogenic aid research and contributing substantially to transformative advancements in the probiotic industry.

## 5 Conclusion

In this study, we verified the longevous and ergogenic effects of TWK10 on *C. elegans* and performed a comprehensive analysis of mass-produced TWK10 in *C. elegans*, mice, and clinical trials. TWK10 administration significantly prolonged lifespan outperforming LGG and ATCC 14917^T^, and specially increased muscle mass, locomotion and glycogen storage on adult *C. elegans*. Four batches of mass-produced TWK10 showed consistent ergogenic effects with laboratory-prepared TWK10 on *C. elegans*. In mice, it significantly improved grip strength, swimming endurance, and glycogen storage in a dose-dependent manner. In healthy male adults, it significant improved the exercise performance and reduced serum lactate and ammonia levels. Both humans and mice showed improved body composition by increasing muscle mass and reducing fat mass. These results demonstrate the longevous and ergogenic effects of TWK10, as well as the consistent ergogenic efficacy of mass-produced TWK10 across *C. elegans*, mice and humans, highlighting *C. elegans* as a reliable tool for relevant probiotic research and industrial applications.

## Funding sources

This research was supported by SYNBIO TECH INC., Kaohsiung, Taiwan.

## CRediT authorship contribution statement

**Jian-Fu Liao:** Writing – original draft, (major lead), Methodology, Investigation, Data curation, Formal analysis, Visualization, Conceptualization. **Chia-Chia Lee:** Writing – review & editing, Investigation, Formal analysis, Conceptualization. **Mon-Chien Lee:** Methodology, (Clinical study), Data curation, Formal analysis. **Han-Yin Hsu:** Writing – original draft, (support), Data curation, Formal analysis, Visualization. **Ming-Fu Wang:** Project administration, (Animal study), Resources, Methodology. **Chi-Chang Huang:** Writing – review & editing, Project administration, (Clinical study), Resources, Methodology. **San-Land Young:** Writing – review & editing, Conceptualization. **Koichi Watanabe:** Writing – review & editing, Conceptualization. **Jin-Seng Lin:** Writing – review & editing, Conceptualization, Project administration, Supervision.

## Declaration of competing interest

The authors declare that they have no known competing financial interests or personal relationships that could have appeared to influence the work reported in this paper.

## Data Availability

Data will be made available on request.
